# Biochar: A Sustainable Solution for Mitigating Greenhouse Gas Emissions and Enhancing Soil Productivity—A Review

**DOI:** 10.1155/sci5/5690423

**Published:** 2026-02-08

**Authors:** Aruna Olasekan Adekiya, Ayibanoa Lekoo Ibaba, Timothy Oyebamiji Ogunbode, Olajire Damilola Adedokun

**Affiliations:** ^1^ Agriculture Programme, College of Agriculture, Engineering and Science, Bowen University, Iwo, Nigeria, bowenuniversity.edu.ng; ^2^ College of Agricultural Sciences, Landmark University, PMB 1001, Omu-Aran, Kwara State, Nigeria, lmu.edu.ng; ^3^ Centre International de Hautes Etudes Mediterraneennes (CIHEAM), Mediterranean Agronomic Institute, Bari, Italy

## Abstract

Greenhouse gases (GHGs) resulting from human activities significantly impact crop production and agricultural sustainability, necessitating innovative solutions to mitigate their effects. One promising approach is employing biochar for GHG mitigation, providing a potential means to offset emissions and enhance crop productivity sustainably. We conducted a comprehensive review by sourcing reputable academic research from various search engines, focusing on terms such as biochar, methane (CH_4_), carbon dioxide (CO_2_), nitrous oxide (N_2_O), GHG, soil organic carbon, agricultural land and cropland. The whole review was divided into three major portions: GHGs, the effect of GHG emission on crop productivity and biochar as an agent of GHG mitigation and further subtopics were designed under each. The review revealed that GHG emissions, including CO_2_, CH_4_ and N_2_O, detrimentally affect crop productivity, posing a serious threat to global food security. Studies demonstrated that biochar aids in mitigating atmospheric CO_2_ by sequestration of C. Studies also demonstrated that biochar can positively influence soil physical properties, such as reducing bulk density and enhancing soil moisture, potentially leading to a decrease in soil N_2_O emissions. The decrease in soil N_2_O emissions was due to the maintenance of optimal oxygen levels in the soil by biochar. Biochar has been utilized to mitigate methane (CH_4_) emissions. The reduction in CH_4_ due to biochar can be linked to the inhibitory effect of biochar chemicals on soil methanogens. However, further research and widespread adoption of biochar use are imperative to fully realize its global potential.

## 1. Introduction

In recent decades, the issue of climate change has emerged as a pressing concern globally, with greenhouse gas emissions (GHGs) being a significant contributor to this phenomenon [1, 2]. Among the various sectors impacted by these emissions, agriculture stands as a critical player, both as a source and a potential mitigator of GHGs [3, 4]. GHGs, including carbon dioxide, methane and nitrous oxide [5], play a pivotal role in climate change due to their heat‐trapping properties [6], directly affecting the Earth’s temperature and weather patterns [7]. Although CH_4_ and N_2_O are emitted in lower quantities than CO_2_, their global warming potentials (GWPs) over a 100‐year horizon are approximately 28 and 273 times that of CO_2_, respectively [8, 9].

Numerous lines of evidence support the assertion that human actions have been the primary driver of global warming since the early 20th century [10]. While natural factors like solar radiation variations, volcanic activity, orbital shifts and the carbon cycle also influence Earth’s radiation balance [11], the predominant impact since the late 1700s has been the consistent elevation of GHG concentrations due to human activities [12]. For example, the emissions of GHGs such as CO_2_ and N_2_O have been reported to occur during dung decomposition [13].

This surge in concentrations of GHG emissions is inducing warming effects and exerting influence over diverse aspects of the climate, including surface and ocean temperatures, precipitation patterns and sea levels [14]. The repercussions of climate change extend to human health, agriculture, water resources, forests, wildlife and coastal regions, rendering them all vulnerable [10]. These gases, predominantly released through human activities, profoundly influence crop production and agricultural sustainability.

It is now widely accepted that agriculture is the main source of anthropogenic N_2_O [8, 15, 16]. Agriculture contributes to 60% of the global N_2_O emissions [17]. Agricultural soils are recognized as the major source of atmospheric N_2_O, globally contributing 1.7–4.8 Tg N yr^−1^ [18]. Agriculture accounts for nearly 12% of global anthropogenic GHG emissions [19]. The concentration of carbon dioxide (CO_2_) in the atmosphere has continued to rise and is now nearly 100 parts per million higher than it was before the industrial revolution [20].

Based on the aforementioned points, it is imperative to address GHG emissions from agricultural soils, and one proposed method is the use of biochar [21, 22]. Biochar, created through pyrolysis of organic materials at high temperatures in the absence of oxygen, possesses key characteristics such as alkali pH, carbon‐rich composition, large surface area and high porosity, making it a suitable soil amendment [23]. Leveraging its physical and chemical properties, biochar application is advocated as a potential approach to enhance soil quality, boost crop yield, mitigate GHG emissions and promote soil carbon sequestration [24].

Although numerous studies have demonstrated the potential of biochar to improve soil quality, enhance microbial activity and reduce GHG emissions, the existing evidence remains fragmented and often inconsistent. For example, Kumar et al. [25] explored biochar’s role as a catalyst/support in advanced oxidation processes but did not deeply link that to soil‐based GHG mitigation within cropping systems. Meanwhile, Sharma et al. [25] documented improvements in soil physical, chemical and biological properties by enhancing soil structure [26], moisture retention, cation exchange capacity (CEC) and nutrient availability, while also stimulating beneficial microbial activity with biochar, and Ralebitso‐Senior and Orr [27] analysed how biochar influences microbial communities. However, each of these focused on isolated effects rather than the integrated pathway connecting soil‐health enhancements to GHG flux reductions. Similarly, Ambika et al. [28] addressed engineered biochar for Cr(VI) remediation, and Iboko et al. [29] conducted a meta‐analysis of biochar + nitrogen fertilizer effects on GHG emissions—but systematic linkage of these outcomes with productivity gains across agroecosystems remains limited. Moreover, variations in biochar feedstock, pyrolysis conditions, soil types and climatic environments have led to contradictory findings, limiting the generalization of results. Long‐term field data, particularly from tropical and sub‐Saharan agricultural systems, are also scarce, while interactions between biochar and other management practices such as fertilizer application and tillage remain poorly understood. Consequently, there is a need for a comprehensive synthesis that bridges these disciplinary and contextual gaps. This review therefore aims to critically evaluate existing literature to elucidate how and under what conditions biochar serves as a sustainable solution for mitigating GHG emissions and enhancing soil productivity, with emphasis on mechanisms, environmental contexts and implications for climate‐smart agriculture.

Therefore, the overall objective of this review is to critically evaluate the potential of biochar as a sustainable solution for mitigating GHG emissions while enhancing soil productivity across agricultural systems.

### 1.1. GHGs

#### 1.1.1. Methane (CH4)

Methane (CH_4_) is a potent GHG with a GWP approximately 27–30 times stronger than CO_2_ over a 100‐year horizon [8, 10]. Its atmospheric concentration has risen steeply in recent decades, reaching approximately 1875 ppb—the highest in at least 800,000 years and about 2.5 times preindustrial levels [30, 31]. The recent acceleration in CH_4_ accumulation has been attributed not only to human activities but also to increasing emissions from natural wetlands driven by climate warming [32], underscoring its sensitivity to climatic feedback.

Anthropogenic activities remain the dominant source of CH_4_, with fossil fuel extraction, livestock production and croplands jointly contributing roughly 50% of global emissions [33, 34]. Agricultural soils and management practices play a key role in shaping CH_4_ dynamics. Nitrogen fertilizers, for instance, influence CH_4_ emissions by altering soil redox balance and microbial processes, stimulating methanogenesis while inhibiting methanotrophic activity [35–37]. Excessive nitrogen rates can exacerbate CH_4_ fluxes, particularly in flooded environments.

Rice paddies remain the largest anthropogenic source of agricultural CH_4_, emitting substantial amounts under anaerobic soil conditions [38, 39]. With climate change intensifying heat and rainfall variability, rice systems face dual challenges: declining productivity and rising CH_4_ emissions. Globally, rice cultivation is estimated to account for approximately 10%–12% of anthropogenic CH_4_ emissions, and up to 18% when broader farm‐level sources are included [40, 41]. Warmer temperatures and prolonged flooding further enhance methanogenic pathways, raising concerns for tropical and Asian rice‐growing regions.

Recent studies have introduced both refined and transformative mitigation options. Proven field‐level interventions include alternate wetting and drying (AWD), which can cut CH_4_ emissions by 30%–70% without yield penalties [39, 42]. Precision fertilization, nitrification inhibitors, deep placement of urea and integrated nutrient management improve nitrogen‐use efficiency while reducing CH_4_ generation [43]. The deployment of low‐emission or methane‐suppressing rice cultivar breeding lines with modified root exudation and rhizosphere microbiomes has gained traction in recent CH_4_ mitigation research [44]. Advanced biological and soil‐based interventions are also emerging. These include the deliberate enhancement of anaerobic methanotrophic archaea to boost in‐soil CH_4_ oxidation, the use of biochar and organic amendments to suppress methanogenesis and the adoption of climate‐adaptive cultivation systems that reduce flooding durations and improve soil aeration [43, 45]. Collectively, these innovations highlight the increasing potential for integrating microbial ecology, soil science and precision agriculture to sustainably curb methane emissions in crop‐based systems.

#### 1.1.2. Carbon Dioxide (CO_2_)

CO_2_ is the primary GHG, with levels rising from 280 ppm (preindustrial) to 415 ppm today [46]. Human activities—fossil fuel combustion and deforestation—drive these increases. Agriculture, forestry and land use account for ∼24% of CO_2_ emissions [47]. Deforestation, reducing carbon sinks, exacerbates emissions [48].

**Figure 1:**
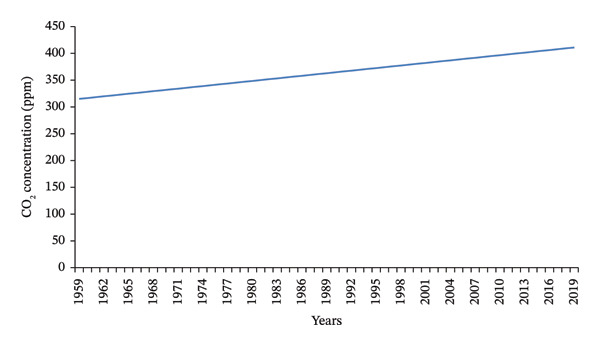
The increase in CO_2_ concentration in the atmosphere (source [49]).

The concentration of carbon dioxide (CO_2_) in the air rose to 411.43 parts per million (ppm) in 2019 from 315.98 ppm in 1959, as depicted in Figure 1 [50]. Scientific estimates suggest that the combined impact of elevated CO_2_ levels and positive water feedback could lead to a 3°C–5°C increase in the average global surface temperature by the Year 2100 [47]. Manure application, while enhancing soil fertility, contributes to CO_2_ emissions via microbial decomposition and anaerobic processes [51]. Improper manure storage further intensifies emissions [52].

#### 1.1.3. Nitrous Oxide (N_2_O)

Though less abundant, N_2_O is 300 times more potent than CO_2_ [53] and constitutes ∼6% of global GHG emissions [54]. Agricultural soils contribute ∼78% of anthropogenic N_2_O emissions [55]. Synthetic fertilizers and manure accelerate N_2_O release through microbial processes [56], with significant implications for ozone depletion [57].

### 1.2. Effect of GHG Emissions on Crop Productivity

GHG emissions, including carbon dioxide (CO_2_), methane (CH_4_) and nitrous oxide (N_2_O), significantly threaten global food security by altering climate conditions essential for crop productivity.

#### 1.2.1. Carbon Dioxide and Crop Growth

Elevated atmospheric CO_2_ can enhance photosynthesis, potentially increasing crop yields. However, this effect varies across species and may be offset by other climate stressors such as temperature fluctuations, ozone alterations and nutrient limitations [58]. Increased CO_2_ also reduces the protein and nitrogen content in crops like alfalfa and soybean, lowering forage quality for livestock [58]. Additionally, C3 plants experience decreased zinc, iron and protein concentrations under elevated CO_2_, affecting human nutrition [59, 60].

#### 1.2.2. Temperature Rise, Heat Stress and Growth Disruptions

Global warming induced by GHGs leads to higher average temperatures, causing heat stress that disrupts photosynthesis, flowering and fruit formation. Elevated temperatures reduce the efficiency of Rubisco, a key enzyme in carbon fixation [61, 62]. Heat stress also damages thylakoid membranes, impairing photosynthesis [63, 64]. Additionally, high temperatures decrease pollen viability and cause floral abnormalities, reducing reproductive success [65, 66].

**Figure 2:**
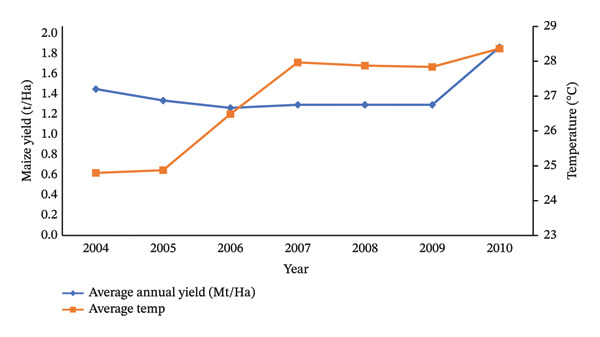
Relationship between total average annual temperature (°C) and annual maize yield (t/Ha) (sources: [67]).

In Figure 2, the correlation between the average annual temperature and the yearly maize yield from 2004 to 2010 in the research location is illustrated. In 2004 and 2005, as the temperature rose from 24.79°C to 24.87°C, respectively, the maize yield decreased from 1.4 to 1.29 t/Ha. This trend continued into 2006, with a temperature increase to 26.48°C resulting in a further decline in maize yield to 1.22 t/Ha.

Evapotranspiration rises with increasing temperatures, exacerbated by prolonged droughts [68]. Heat stress dehydrates rice plants, leading to crop loss [69]. Higher temperatures extend the warm season, shortening crop cycles and reducing maize yields by affecting pollination and seed germination [70]. A 1°C rise in temperature can decrease maize yield by 10% [71]. Climate change is also expected to extend growing seasons, potentially disrupting crop adaptation and yield [72, 73].

#### 1.2.3. Changes in Precipitation Patterns

**Figure 3:**
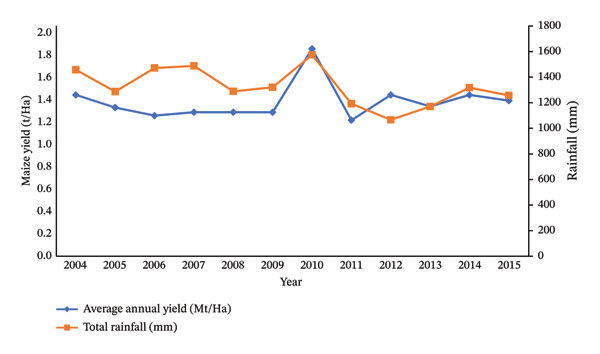
Relationship between annual rainfall (mm) and annual maize yield (t/Ha) (sources: [67]).

Climate change alters rainfall patterns, leading to more intense or prolonged droughts. These shifts disrupt crop cycles, affecting germination, water availability and nutrient uptake. With over 80% of global crop yields dependent on rainfall, such changes have critical implications [74]. Figure 3 illustrates the correlation between yearly precipitation and maize crop yield from 2004 to 2015 within the specified study region. In 2004, there was a reduction in rainfall from 1456.7 to 1286.2 mm, which coincided with a decrease in maize yield from 1.4 to 1.29 t/Ha. This pattern persisted into 2005. However, a notable shift occurred in 2006 when maize yield dropped to 1.22 t/Ha, despite a significant rise in rainfall to 1470.7 mm. Between 2006 and 2007, there was a slight increase in rainfall, and correspondingly, maize yield saw a slight rise. This trend remained consistent in 2008 and 2009, maintaining a steady maize yield of 1.25 t/Ha.

Insufficient soil moisture weakens plant resilience, increasing susceptibility to pests and diseases [75]. Conversely, excessive rainfall in regions like the Atlantic coast and European mountains leads to yield losses, poor soil workability and reduced operational days for machinery [74]. In Nigeria, extreme weather events cause crop production fluctuations, affecting prices and food security [76]. Similarly, monsoon‐induced floods in Thailand threaten agricultural output [77].

#### 1.2.4. Pests, Diseases and Climate Change

Rising temperatures favour pests and diseases, altering their distribution and increasing infestations [78]. Climate change drives insect population dynamics, worsening crop losses [79]. Warmer conditions create ecological niches for pest migration [80] and facilitate plant infections by altering pathogen evolution and host–pathogen interactions [81, 82]. Elevated CO_2_ intensifies crop diseases, including powdery mildew in cucurbits and wheat blight [83, 84]. Additionally, humidity and heat worsen potato blight and oilseed rape canker [85].

#### 1.2.5. Water Resources and Soil Fertility

Climate change disrupts precipitation and evaporation cycles, reducing irrigation water availability and increasing water pollution risks [86]. Saltwater intrusion from rising sea levels damages water infrastructure [86]. Soil organic matter decomposition releases dissolved organic matter, impacting water quality [87]. Climate‐driven soil degradation accelerates erosion, nutrient leaching and loss of soil organic matter, increasing compaction and reducing fertility [88–90]. Higher temperatures intensify organic matter decomposition, lowering CEC and leading to soil acidification [91, 92].

### 1.3. Biochar Effects on Soil Properties

The application of biochar to agricultural soils has been shown to influence a range of physical, chemical and biological soil properties, thereby potentially enhancing soil productivity and contributing to GHG mitigation.

#### 1.3.1. Soil Physical Properties

Studies have shown that biochar application can improve soil structure, reduce bulk density and improve aggregate stability. For example, Ebido et al. [93] found that rice husk biochar (RHB) added to a coarse‐textured Ultisol increased soil organic carbon (SOC) and improved aggregate stability by up to ∼17% at the highest rate (≈60 t ha^−1^) compared to the unfertilized control. Improvements in porosity and water‐holding capacity have also been reported, particularly in sandy soils: biochar’s porous structure increases spaces for water retention and microbial habitat, thus improving retention and movement of water in the soil profile. More broadly, reviews show that biochar tends to reduce soil bulk density, increase porosity and improve aggregate stability/mean weight diameter (MWD) [94]. For example, an 8‐year field experiment in northeast China in a Mollisol found aggregate stability increased by ∼10.9%–23.5% with biochar application; the authors attribute this to the porous structure of biochar and its capacity to serve as binding sites for microaggregates [95]. In one long‐term U.S. no‐till field, application of papermill biochar reduced bulk density from ∼1.40 to ∼1.26 g cm^−3^ and increased aggregate stability by up to ∼67% after 10 years of continuous use [96]. The high specific surface area and porosity of biochar allow for particle–microbe–mineral interactions, enhancing binding of soil particles into aggregates; hydrophilic functional groups on the biochar surface (carboxyl, hydroxyl) improve electrostatic or hydrogen‐bond–mediated particle binding.

In sandy or coarse‐textured soils, biochar’s highly porous matrix offers additional storage of water and enhances plant‐available water content (PAWC). For instance, a meta‐analysis found that coarse‐textured soils amended with biochar had ∼30% greater plant‐available water capacity [97]. Another review summarizes that biochar improved field water‐holding capacity, soil available water content and reduced saturated hydraulic conductivity (sometimes) under certain conditions [98]. The mechanism involved included the fact that biochar provides both macro‐ and micropores; the micropores hold water against gravity, while macropores enhance infiltration and connectivity; increased porosity and improved aggregate structure reduce compaction and improve water movement and retention. However, some caveats remain. In very weathered tropical soils, one study found biochar increased aggregate stability by up to 33% but had no detectable effect on field‐saturated hydraulic conductivity or water‐retention characteristics after 10 months [99].

#### 1.3.2. Soil Chemical Properties

On the chemical side, biochar application exerts multiple beneficial effects on soil fertility and nutrient dynamics. Generally, biochar tends to raise soil pH, particularly when produced from feedstocks at high pyrolysis temperatures (≥ 500°C), owing to the concentration of basic cations such as Ca^2+^, Mg^2+^, K^+^ and Na^+^ in its ash fraction [100]. This liming effect is especially valuable for acidic tropical soils, such as the Alfisols of the Nigerian‐derived savannah, which are often prone to nutrient fixation and aluminium toxicity. For instance, Obalum et al. [101], in their review of Nigerian agroecologies, observed that most locally produced biochars are alkaline (pH > 8) and their application frequently ameliorates soil acidity while enhancing nutrient availability and base saturation. Similarly, Oguntunde et al. [102] reported that the application of maize–stalk biochar at 10–20 t ha^−1^ increased soil pH from 5.3 to 6.8 in a degraded Alfisol in southwest Nigeria, creating a more favourable environment for crop nutrient uptake. Biochar also enhances CEC and nutrient retention through its high surface area, porous structure and abundance of negatively charged functional groups (carboxyl, phenolic and hydroxyl moieties). These characteristics increase the soil’s ability to retain exchangeable cations (NH_4_^+^, K^+^, Ca^2+^, Mg^2+^), thereby reducing nutrient leaching and improving fertilizer‐use efficiency [103]. In tropical and subtropical soils, where leaching losses are common due to high rainfall, the addition of biochar has been shown to significantly enhance nutrient retention and plant nutrient uptake. El‐Naggar et al. [104] applied biochars produced from rice straw, silvergrass residues and umbrella tree to sandy soils and reported CEC increases of 906%, 180% and 130%, respectively, compared to unamended soil.

Moreover, biochar serves as a sink and stabilizing agent for SOC. Its carbon is highly aromatic and recalcitrant, contributing to long‐term carbon sequestration and improving soil organic matter quality [105]. In addition, biochar can physically protect native organic matter by promoting the formation of organo‐mineral complexes and microaggregates, thereby enhancing the stability of SOC [106]. Ebido et al. [93] also reported that RHB addition to an Ultisol significantly increased SOC concentration, supporting the notion that biochar enhances both the labile and stable carbon pools [107]. Furthermore, biochar can modulate nitrogen dynamics by adsorbing ammonium (NH_4_^+^) and nitrate (NO_3_^−^), thus reducing N losses through leaching or volatilization [106]. The retention of these ions not only enhances soil fertility but also minimizes environmental pollution from reactive nitrogen. For instance, Li et al. [96] reported that biochar‐amended soils retained 35% more NH_4_^+^–N and 28% more NO_3_^−^–N compared to unamended soils, leading to improved nitrogen‐use efficiency in soybean systems (Table 1).

**Table 1:** Effects of biochar on soil chemical properties.

Soil chemical property	Main findings	Benefits to soil/crops	Citations
Soil pH (liming effect)	Biochar raises soil pH, especially when produced at ≥ 500°C due to high levels of basic cations (Ca^2+^, Mg^2+^, K^+^, Na^+^). Locally produced Nigerian biochars commonly have pH > 8. Application of maize–stalk biochar (10–20 t ha^−1^) increased soil pH from 5.3 to 6.8 in an Alfisol	Reduces soil acidity, alleviates Al toxicity, improves nutrient availability, enhances root growth and fertilizer‐use efficiency	[101, 102]
Cation exchange capacity (CEC)	Biochar improves CEC due to high surface area, porosity and negatively charged functional groups (carboxyl, phenolic, hydroxyl). In sandy soils, CEC increased by 906%, 180% and 130% using rice straw, silvergrass and umbrella‐tree biochars	Enhances nutrient retention (NH_4_^+^, K^+^, Ca^2+^, Mg^2+^), reduces leaching losses, increases fertilizer‐use efficiency	[103, 104]
Soil organic carbon (SOC)	Biochar contributes highly aromatic, recalcitrant carbon, improving long‐term carbon sequestration. Promotes formation of organo‐mineral complexes and microaggregates. Rice husk biochar increased SOC in Ultisols	Enhances stable and labile carbon pools, improves soil structure, boosts microbial activity and increases soil resilience	[93, 105, 106]
Nitrogen dynamics	Biochar adsorbs NH_4_^+^ and NO_3_^−^, increases soil N retention and reduces leaching and volatilization. Biochar‐amended soils retained 35% more NH_4_^+^–N and 28% more NO_3_^−^–N compared to controls	Improves nitrogen‐use efficiency, reduces fertilizer requirements and lowers environmental pollution from reactive nitrogen	[211, 212]

#### 1.3.3. Soil Biological Properties

On the biological and biochemical side, biochar significantly influences soil microbial communities, enzyme activities and overall biochemical functioning. Through its porous structure, surface chemistry and organic carbon content, biochar creates new microhabitats and energy sources for soil microorganisms [100]. These pores can protect microbes from environmental stress (e.g., desiccation and predation) while providing surfaces for colonization, thereby enhancing microbial abundance and diversity [108]. Biochar’s porous matrix also modifies soil aeration, moisture and nutrient availability, all of which are key determinants of microbial activity. By improving soil moisture retention, biochar stabilizes the habitat for microbial communities during dry periods, particularly in coarse‐textured tropical soils [109]. Furthermore, the sorption of labile organic molecules and toxins on biochar surfaces can buffer microbial processes by reducing substrate loss and toxicity [110]. Biochar amendments often result in increased microbial biomass and enzymatic activity, which in turn accelerate nutrient cycling. For instance, Ebido et al. [93] reported that RHB application in an Ultisol enhanced microbial biomass carbon and increased dehydrogenase and phosphatase activities, reflecting improved biological functioning and nutrient turnover (Table 2).

**Table 2:** Effects of biochar on soil biological properties.

Biological property	Main findings	Benefits to soil and crop productivity	Citations
Microbial habitat formation	Biochar provides porous microhabitats that support microbial colonization and protect microbes from desiccation, predation and temperature stresses. The pores and surfaces act as ecological niches for bacteria and fungi	Enhances microbial abundance, diversity and resilience; promotes stable microbial networks crucial for nutrient cycling	[100, 108]
Microbial biomass and diversity	Biochar increases microbial biomass carbon, microbial diversity and functional group abundance due to improved aeration, moisture and bioavailable carbon	Greater microbial activity enhances soil fertility, nutrient release and organic matter decomposition	[93, 213]
Soil moisture and aeration effects on microbes	Biochar improved water retention stabilizes microbial habitats, especially in sandy tropical soils. Enhanced aeration supports aerobic microbes while reducing stress periods in dry seasons	Sustains microbial functioning under drought; improves decomposition, nutrient mobilization and soil resilience	[109, 214]
Sorption of labile molecules and toxins	Biochar adsorbs labile organic substrates, pesticides and toxic compounds, reducing their mobility and preventing microbial inhibition	Creates a safer and more buffered biochemical environment for microbes, improving enzymatic activity and soil health	[110, 215]
Enzyme activities (e.g., dehydrogenase, phosphatase, urease)	Biochar stimulates soil enzyme activities involved in carbon, nitrogen and phosphorus cycling. Increased dehydrogenase and phosphatase activities reported in Ultisols after rice husk biochar addition	Faster nutrient cycling, improved organic matter turnover and enhanced availability of N, P and C for crops	[93, 216]
Microbial community structure and functional genes	Biochar shifts microbial communities towards beneficial taxa (e.g., plant growth–promoting rhizobacteria, N‐fixers and mycorrhizae). Enhances functional genes related to nitrification, denitrification and carbon metabolism	Improved nutrient‐use efficiency, reduced GHG‐producing microbes (e.g., CH_4_ methanogens), better crop–soil interactions	[96, 217]
Plant–microbe interactions	Biochar enhances root colonization by beneficial microbes and mycorrhizal fungi; reduces pathogen abundance through improved soil environment and sorption of toxins	Improved plant health, reduced disease incidence, enhanced nutrient uptake and root performance	[218, 219]

### 1.4. Biochar as an Agent of GHG Sequestration

At present, crop yield uncertainties and rising GHG emissions have marred the overall productive capacity of agriculture systems, putting future food security targets in jeopardy. Indeed, this peculiar situation emphasizes the importance of transitioning from modern intensive farming to more sustainable agricultural management, which can boost crop productivity while reducing GHG emission.

Biochar, a carbon‐rich charcoal material, is produced by a dry carbonization process, either under the complete or partial absence of O_2_, at high temperatures ranging from 300°C to 1000°C [111]. Globally, biochar has attracted considerable attention as a versatile organic amendment with significant potential for mitigating the global warming effects [112], increasing crop productivity [100] and C‐sequestration [113]. The availability of wide‐ranging feedstock materials, as well as the pyrolysis temperature conditions, can produce biochar of varied physical and structural attributes, including but not limited to mechanical strength, porosity, surface area, particle size, and density and structural complexity [100, 114].

Biochar application can replenish key soil nutrients in low‐fertility soils due to its unique surface charge density, and the predominant negatively charged surfaces of biochar also promote cation adsorption [115, 116]. Since the sources and sinks of three potent GHGs (CO_2_, N_2_O and CH_4_) are major components of the C budget in ecosystems, the inclusion of biochar as a soil amendment is critical because it can sequester C and, more importantly, enable soil to negate anthropogenic CO_2_ emissions [117]. According to estimates, biochar produced from 2.2 Gt of feedstock material can remove up to 0.49 Gt C from the atmosphere each year, implying greater merits for its use as a key climate change mitigation strategy [118].

#### 1.4.1. Effect of Biochar on CO_2_ Sequestration

Carbon sequestration is a process by which atmospheric CO_2_ is captured and stored to prevent it from being emitted into the atmosphere [119]. It is essential that the carbon is transferred to a passive carbon pool, i.e., stable or inert, in order to decrease C emission to the atmosphere. Transferring even a small amount of the carbon that cycles between the atmosphere and plants to a much slower biochar cycle would impact greatly on atmospheric CO_2_ concentration. Biochar is biologically and chemically more stable than the original carbon form, due to its molecular structure and its origins. Furthermore, it is difficult for the sequestered carbon to be released as CO_2_, making this a good method for carbon sequestration [120].

Biochar plays a crucial role in carbon sequestration by capturing and storing carbon in a stable form within the soil. The process involves multiple mechanisms, including:

1. Pyrolysis and Carbon Stabilization: Biochar is produced through pyrolysis, a process that involves the thermal decomposition of biomass in an oxygen‐limited environment [121]. During pyrolysis, labile carbon (e.g., carbohydrates, proteins) is converted into recalcitrant carbon, forming aromatic structures that resist microbial degradation [122]. A comprehensive 8‐year study examined the decomposition of biochar derived from ryegrass using compound‐specific 14C analysis. The results demonstrated an exceptionally slow decomposition rate, with the biochar losing only 7 × 10^−4^% of its carbon content per day under optimal conditions [123]. This suggests that nearly 400 years would be required for just a 1% reduction in its carbon content. This stabilized carbon remains in the soil for hundreds to thousands of years, preventing its rapid decomposition and release as CO_2_ back into the atmosphere [124]. This longevity ensures that the sequestered carbon remains stored in the soil, providing a reliable and long‐term solution for carbon sequestration;
2. Reduced Microbial Respiration and Mineralization: Biochar has a high carbon‐to‐nitrogen (C:N) ratio, which limits its decomposition by microbes [125]. Its complex chemical structure inhibits microbial respiration and slows down carbon mineralization, reducing CO_2_ emissions [126]. Biochar amendments possess the capacity to modify enzymatic activity through their influence on microbial community composition and metabolic processes, thereby instigating alterations in the rates of organic matter decomposition and subsequent CO_2_ sequestration [127]. It also promotes microbial interactions that enhance carbon stabilization by forming microbial biofilms on its surface [128].
3. Soil Carbon Protection via Adsorption and Aggregate Formation: Biochar adsorbs dissolved organic carbon (DOC), preventing its leaching and subsequent oxidation into CO_2_ [129]. The potential for biochar (charcoal produced from pyrolysed filtercake) to mitigate carbon and nutrient leaching in a cultivated Brazilian Ferralsol after vinasse application was evaluated [129]. Results of their experiment revealed that biochar‐amended soil preferentially retained high‐molecular‐weight, humic‐like DOC species, as revealed by fluorescence spectroscopy and optical indices. Thus, biochar amendments in vinasse application areas may decrease carbon leaching.
4. Enhanced Plant Growth and Photosynthetic Carbon Fixation: By improving soil fertility, water retention and CEC, biochar enhances plant growth [130]. This leads to greater CO_2_ uptake by plants through photosynthesis, thereby increasing biomass production. Some of this biomass is eventually returned to the soil as organic matter, further contributing to long‐term carbon sequestration.

Several studies have been carried out on the effect of biochar on the sequestration of atmospheric CO_2_, with contrasting results. Some studies report a reduction in CO_2_ emissions; Sun et al. [130] reported that the application of biochar at the rate of 30 t/ha reduced CO_2_ emissions by 31.5% from a pine forest soil. Application of biochar significantly reduced CO_2_ emissions as reported by Qiao and Wu [131]. Gui et al. [132] in their work aimed to explore if the extra sorption of carbon dioxide (CO_2_) exists in the biochar‐amended soil. They put biochar and mineral‐rich biochar into soils to perform laboratory CO_2_ sorption experiments. Their results demonstrate that all biochars increased soil carbon storage and meanwhile further sorb CO_2_ for more carbon sequestration. It has been reported that the biochar technology could deliver emission reductions of 3.4∼6.3 Pg CO_2_ annually and persist in soil for hundreds to thousands of years [133, 134]. Sheng et al. [135] also found suppression of total CO_2_ release in both 500°C‐ and 700°C‐derived biochar‐amended soils. By using a generalized framework for quantifying, the potential contribution biochar can make towards achieving national carbon emissions reduction goals, assuming use of only sustainably supplied biomass, that is, residues from existing agricultural, livestock, forestry and wastewater treatment operations [136]. Results showed that biochar can play a role in worldwide CDR strategies, with carbon dioxide removal potential of 6.23 ± 0.24% of total GHG emissions in the 155 countries covered based on 2020 data over a 100‐year timeframe, and more than 10% of national emissions in 28 countries.

Others report an opposite effect; a field experiment by Zhang et al. [137] reported that wheat straw biochar had significantly increased CO_2_ emissions by 12%. Hawthorne et al. [138] found that CO_2_ emission from Douglas‐fir forest soil was higher under biochar application at the rate of 10% compared to the rate of 1%. Some studies report no effect at all. Fidel et al. [139] observed no reduction in CO_2_ emissions after application of biochar in a field study with four cropping systems (continuous corn, switchgrass, low diversity grass mix and high diversity grass–forb mix). Cropping system however had a significant effect in the field study, with soils in grass and grass–forb cropping systems emitting more CO_2_ than the continuous corn cropping system. Another field experiment in a pasture ecosystem showed no significant effects of biochar amendment on soil CO_2_ emissions [140].

These contrasting effects of biochar on sequestration of atmospheric CO_2_ could be because biochar influences soil total CO_2_ emissions differently depending on biochar type, soil type and experimental design [135, 141–144]. Cely et al. [145] found that biochar derived from wood chips exhibited a negative priming effect in soils, whereas biochar produced from a mixture of paper sludge and wheat husk induced a positive priming effect. These differences may be attributed to variations in biochar properties, including carbon content, carbon aromaticity, volatile matter, fixed carbon, easily oxidized organic carbon, metal content and phenolic compounds, as well as surface characteristics. Their study indicated that biochar application increased soil CO_2_ emissions by approximately 25%.

#### 1.4.2. Effect of Biochar on N_2_O Sequestration

The major source of N_2_O emissions from agricultural soils is the application of synthetic nitrogen (N) fertilizer, as highlighted by Wang et al. [144]. Studies demonstrate that biochar can positively influence soil physical properties, such as reducing bulk density [146] and enhancing soil moisture [147], potentially leading to a decrease in soil N_2_O emissions. The global trend towards adopting no‐tillage and reduced tillage practices is driven by their demonstrated benefits in enhancing soil organic matter status, structural condition and water regime [148, 149]. Extensive studies indicate that reduced tillage induces a significant alteration in soil structure, increasing porosity and decreasing bulk density over the long term [150]. The transition from traditional mouldboard ploughing to no‐tillage has been associated with a decrease in N_2_O emissions [151].

Literature acknowledges the potential of soil compaction to elevate N_2_O emissions from agricultural soils. For instance, Hu et al. [152] conducted a review on the effects of soil compaction on productivity and the environment, using New Zealand as a case study. Their findings suggest that the role of soil compaction in explaining variability in N_2_O emissions remains unclear. Ruser et al. [153] reported a 20% increase in the bulk density of fine silty soil, leading to a rise in N_2_O emission. Studies examining N_2_O emissions in different contexts further support these observations. In a Czech Republic cattle overwintering area, Simek et al. [154] observed higher N_2_O emissions in trampled areas, though statistical significance was not achieved, attributed to high spatial variability. Similarly, a study in Scotland simulated trampling in a wet dairy pasture soil, revealing a threefold increase in N_2_O emissions [155]. In New Zealand, van der Weerden and Styles [156] and van der Weerden et al. [157] noted elevated N_2_O fluxes from compacted treatments after urine application in pasture on silt loam soil. In oak forests, Goutal et al. [158] found higher N_2_O production in trafficked plots compared to the control treatment, particularly below 0.3 m depth, where soil air‐filled porosity was significantly reduced. These reactions were because compaction‐induced changes in the pore system, as highlighted by Dörner and Horn [159], negatively impact pore size, tortuosity and connectivity. This alteration in the pore structure directly influences fluid transport in soil [160, 161], potentially leading to anaerobic conditions and altered soil processes [162, 163]. In summary, limited pore continuity and reduced gas transport capacity within and between aggregates influence N_2_O production, consumption and transport to the soil surface, as outlined by Ball [164].

Rondon et al. [165] were among the first to report a reduction in soil N_2_O emissions following biochar application. Conducted in a low‐fertility oxisol Colombian savannah, their study revealed a reduction of up to 50% for soybeans and up to 80% for grass in N_2_O emissions. Additionally, Liu et al. [166] conducted a laboratory study on two types of biochar derived from rice straw and dairy manure, finding a correlation between reduced copy numbers of the monooxygenase gene amoA and the nitrite reductase gene nirS (genes responsible for nitrification and denitrification) and a subsequent reduction in N_2_O emissions when biochar was applied.

In a study assessing the impact of biochar on soil GHG emissions at both laboratory and field scales, Fidel et al. [139] observed a 27% suppression of N_2_O emissions in a corn cropping system. However, they noted no significant effect at the laboratory scale across varying soil temperature and moisture levels. This disparity in N_2_O emission results between laboratory and field‐scale studies emphasizes that laboratory experiments may not reliably predict the impact of biochar at the field scale. Yanai et al. [147] conducted a brief laboratory incubation study, applying municipal biowaste‐derived biochar at a rate of 180 tonnes per hectare. They observed a noteworthy reduction in N_2_O emissions within a wetted volcanic ash soil. Correspondingly, Zhang et al. [167] demonstrated a significant decline in total N_2_O emissions in a hydroagric stagnic anthrosol by 40%–51% and 21%–28% after the addition of biochar (created via slow pyrolysis of wheat straw at 350°C–550°C at a rate of 40 tonnes per hectare compared to control treatments, with or without N‐fertilizer, respectively). This aligns with the results of Sarkhot et al. [168], who also found a 26% reduction in cumulative N_2_O flux when using dairy manure‐derived biochar (DBC).

Furthermore, Cayuela et al. [169] conducted a comprehensive meta‐analysis encompassing both short‐ and long‐term studies evaluating the impact of biochar on N_2_O emissions. They observed a 54% reduction in soil N_2_O emission under controlled laboratory conditions and a 28% reduction in field conditions. Their meta‐analysis highlighted significant factors influencing N_2_O emissions, including the type of feedstock used for biochar production, pyrolysis conditions and the properties of the resulting biochar. Additionally, they found a direct relationship between the reduction of N_2_O emissions and the application rates of biochar [169]. The study also proposed that interactions among biochar, soil texture and nitrogen fertilizer form play a pivotal role in affecting soil N_2_O emissions [169].

The reduction of soil nitrous oxide (N_2_O) emissions by biochar is attributed to several mechanisms, such as: (1) Improved Soil Aeration and Reduced Denitrification: Biochar enhances soil structure, increasing porosity and aeration [170], which promotes oxygen diffusion [171]. This reduces anaerobic conditions, limiting denitrification, a major source of N_2_O emissions. The availability of oxygen to soil microorganisms via water‐filled pore space (WFPS) depends on soil moisture and aeration, ultimately influencing the activities of nitrifiers and denitrifiers. A study by Bateman and Baggs [172] investigated the contributions of nitrification and denitrification to N_2_O emissions at different WFPS levels. The findings revealed that nitrification was the primary cause of N_2_O generation at 35%–60% WFPS, while denitrification dominated at 70% WFPS and higher [172]. (2) Adsorption and Retention of Nitrogen Compounds: Biochar has a high surface area and CEC, allowing it to adsorb ammonium (NH_4_^+^) and nitrate (NO_3_^−^) [173, 174]. By retaining nitrogen, biochar reduces substrate availability for nitrification and denitrification, thereby lowering N_2_O production [175, 176]. Zhong et al. [177] investigated the potential for N_2_O production through bacterial and fungal nitrification and denitrification in both rhizosphere and nonrhizosphere soils, along with the abundance of microbial genes associated with these processes. Their findings revealed that inorganic fertilizers and biochar significantly influenced N_2_O production potential and gene abundance. They concluded that partially replacing inorganic fertilizers with biochar could mitigate N_2_O emissions by reducing bacterial nitrification and denitrification. (3) Altering Microbial Communities: Biochar influences soil microbial dynamics, particularly by suppressing denitrifiers responsible for N_2_O production. Case et al. [178] investigated the effect of biochar on soil N_2_O emissions and N cycling processes by quantifying soil N immobilization, denitrification, nitrification and mineralization rates using ^15^N pool dilution techniques and the FLUAZ numerical calculation model. They examined whether biochar amendment affected N_2_O emissions and the availability and transformations of N in soils. Results showed that biochar suppressed cumulative soil N_2_O production by 91% in near‐saturated, fertilized soils. Cumulative denitrification was reduced by 37%, which accounted for 85%–95% of soil N_2_O emissions. Biochar also enhances N_2_O‐reducing bacteria, which convert N_2_O to harmless N_2_ gas [179]. (4) pH Modification and Inhibition of N_2_O Formation: Biochar tends to increase soil pH [114], which can reduce nitrification rates (lowering NO_3_^−^ availability). In the study of Ippolito et al. [180] in a hardwood‐based fast pyrolysis in which biochar was applied (0 wt.%, 1 wt.%, 2 wt.% and 10 wt.%) to calcareous soil, it was reported that biochar at higher applications dramatically lowers soil NO^−^_3_−N concentrations and prevents NO^−^_3_−N from accumulating over time. An incubation experiment was performed on the salt‐affected soil collected from a three‐year consecutive experiment at biochar application gradients of 7.5, 15 and 30 t⋅ha^−1^ and under nitrogen (N) fertilization [181]. Biochar addition inhibited nitrification in salt‐affected irrigation‐silting soil by shifting the community structures of ammonia‐oxidizing bacteria and ammonia‐oxidizing archaea and reducing the relative abundance of dominant functional ammonia‐oxidizers, such as *Nitrosospira*, *Nitrosomonas* and *Nitrosopumilus*. Biochar also tends to increase soil pH, which can shift denitrification pathways towards complete reduction of N_2_O to N_2_ gas, minimizing emissions. Biochar was found to suppress N_2_O and NO emissions by altering soil pH during denitrification [182]. Using acid acrisols and two biochar types, the study found that biochar’s alkalizing effect, not labile carbon, influenced product stoichiometry. Acid‐leached biochar lost their suppression ability, confirming pH’s critical role in denitrification product dynamics. In order to study the influence of biochar addition on N_2_O emissions from soils with different pH levels [183], a 40‐day incubation experiment was carried out, and four treatments (control, nitrogen fertilizer application, biochar amendment and N plus biochar amendment) were set up separately in soils with three different natural pH levels (acidic vegetable soil, neutral rice soil and alkaline soil). Results showed that adding biochar significantly decreased N_2_O emissions by 20.8% and 47.6% in acidic vegetable soil for both N and no‐N addition treatments, respectively. Thus, biochar amendment could be used as an effective management practice for mitigating N_2_O emissions from acidic and alkaline soils (Table 3).

**Table 3:** Effect of biochar on N_2_O sequestration.

Theme/factor	Main findings	Benefits/implications	Citations
Synthetic N‐fertilizer	Major driver of N_2_O emissions in agricultural soils	Highlights need for improved N management strategies	[220]
Biochar—physical properties	Biochar reduces bulk density and improves moisture retention	Enhances aeration and reduces N_2_O generation potential	[146, 147]
Reduced/no‐tillage	Improves SOM, soil structure and porosity; lowers bulk density; and decreases N_2_O emissions compared to conventional ploughing	Supports climate‐smart, conservation agriculture practices	[148–151]
Soil compaction	Higher bulk density alters pore connectivity; increases N_2_O emissions	Avoiding compaction reduces denitrification‐induced N_2_O flux	[152, 153]
Livestock trampling studies	Trampled and compacted areas show higher N_2_O emissions (sometimes with high spatial variability)	Highlights the risk of emissions hotspots in grazed systems	[154, 155]
Compaction + urine deposition	Compacted pasture soils show intensified N_2_O flux after urine application	Importance of manure/urine management in grazing systems	[156, 157]
Forest and woodland compaction	Trafficked plots in oak forests show increased N_2_O production, especially at depth	Forest soil trafficking can influence greenhouse gas dynamics	[158]
Mechanistic understanding of compaction	Reduces pore size, tortuosity, connectivity; restricts gas transport; promotes anaerobic zones	Identifies structural drivers of N_2_O variability; guides soil management reforms	[159–164]
Early evidence of biochar mitigation	Biochar reduced N_2_O by up to 50%–80% in oxisols	Strong evidence for mitigation in low‐fertility tropical soils	[155]
Biochar impacts on microbial genes	Reduces amoA and nirS gene copies linked to nitrification/denitrification	Demonstrates microbial mechanism behind N_2_O reduction	[166]
Biochar + no‐till systems	No‐tillage + biochar significantly lower N_2_O emissions vs. conventional tillage	Integrated strategies amplify mitigation effects	[221]
High‐rate biochar applications	Substantial N_2_O reductions in volcanic ash soils at 180 t ha^−1^	Shows the sensitivity of N_2_O response in certain soil types	[147]
Wheat‐straw biochar	Reduced N_2_O by 40%–51% (with N) and 21%–28% (without N)	Effective across fertilized and unfertilized systems	[167]
Manure‐derived biochar	26% reduction in cumulative N_2_O emissions	Demonstrates the value of recycled organic waste biochars	[168]
Meta‐analysis (biochar)	N_2_O emissions reduced by 54% (lab) and 28% (field). Effects vary by feedstock, pyrolysis and rate	Strong global evidence base for biochar as a mitigation tool	[169]
Improved aeration mechanism	Biochar improves porosity, oxygen diffusion; reduces anaerobic hotspots	Limits denitrification and associated N_2_O production	[170–172]
N retention mechanism	Adsorbs NH_4_^+^ and NO_3_^−^, decreasing substrates for N_2_O‐producing pathways	Enhances nitrogen‐use efficiency	[173–177]
Microbial community shifts	Biochar suppresses denitrifiers; increases N_2_O‐reducing bacteria; reduces cumulative denitrification	Direct biological control of N_2_O emissions	[178, 179]
pH modification mechanism	Biochar increases soil pH, reducing nitrification rates and shifting denitrification towards complete reduction to N_2_	Particularly effective in acidic or salt‐affected soils	[148, 180–183]

#### 1.4.3. Effect of Biochar on CH_4_ Sequestration

Biochar has been utilized to mitigate methane (CH_4_) emissions. In a study by Yu et al. [184], a notable reduction in CH_4_ emissions from forest soils was observed with the incorporation of 10% w/w chicken manure biochar. Similarly, Xiao et al. [185] demonstrated that biochar significantly enhanced CH_4_ uptake in a chestnut plantation in China, irrespective of the application rate. The increase in CH_4_ uptake within the soil can be attributed to biochar‐induced elevation of soil pH, promoting the growth of methanotrophs [186, 187]. Additionally, biochar application leads to a reduction in soil bulk density and an increase in soil porosity, facilitating CH_4_ oxidation and uptake by soil microbes [188, 189]. The reduction in CH_4_ due to biochar can be linked to the inhibitory effect of biochar chemicals on soil methanotrophs [190]. The porous structure of biochar provides new habitats for soil microbes, enhancing CH_4_ uptake by methanotrophs more than CH_4_ production, owing to improved soil aeration [189]. A study by Karhu et al. [189] highlighted that CH_4_ uptake increased in biochar‐amended soil due to enhanced soil aeration and improved CH_4_ diffusion through the soil profile. From a laboratory experiment, Liu et al. [21] found that CH_4_ emissions from paddy soil amended with bamboo char and straw char at 2.5% application rate were reduced by 51.1% and 91.2% in 49 days.

Lee et al. [191] reported a global meta‐analysis conducted regarding the effectiveness of biochar. The study by Jeffery et al. [187] demonstrated that biochar application effectively mitigates methane (CH_4_) emissions while enhancing SOC and crop yield. Their quantitative meta‐analysis revealed that biochar substantially reduces CH_4_ emissions, especially in flooded (paddy) fields and acidic soils where flooding occurs as part of field management. Rajalekshmi et al. [192] found that the application of RHB in wetland Ultisol significantly enhanced soil carbon content and increased carbon accumulation in rice crops. The study revealed that RHB contributed substantial refractory carbon to the soil, improving its nutrient status and productivity. Importantly, RHB application led to a notable 50%–60% reduction in methane (CH_4_) emissions compared to farmyard manure (FYM), highlighting its effectiveness in mitigating GHG emissions while enhancing soil and crop quality.

An experiment was conducted to evaluate the potential of mangrove tree wood (*Rhizophora apiculata*) biochar on CH_4_ mitigation, soil properties and the productivity of rice cultivated in a clay loam soil in Thailand [193]. The treatment biochar alone significantly reduced cumulative methane (CH_4_) emissions compared to the nonamended control: 21.1% in the first season, 24.9% in the second season. The treatment biochar + fertilizer also produced lower CH_4_ emissions than the fertilizer‐alone treatment. A 2‐year field study conducted in the sandy loam soils of Inner Mongolia, China, examined the effects of corn residue–derived biochar on GHG emissions, SOC, and GWP under film‐mulched, drip‐irrigated maize production [194]. Corn residue–derived biochar, particularly at 15–30 t ha^−1^, effectively reduced CH_4_ and N_2_O emissions, improved SOC storage and lowered net GWP, demonstrating its potential as a climate mitigation and soil‐improvement strategy in semiarid sandy loam soils under drip‐irrigated maize production in northern China.

Biochar reduces methane (CH_4_) emissions from the soil through several mechanisms: (1) Enhanced Aeration and Redox Potential: Biochar improves soil structure, increasing aeration and oxygen diffusion [170]. This promotes aerobic microbial activity, suppressing methanogenic (CH_4_‐producing) archaea that thrive in anaerobic conditions. The effect of biochar addition on CH_4_ emissions, and the abundance and community composition of methanogens and methanotrophs over two rice cultivation seasons were studied [195]. Biochar application decreased CH_4_ emissions by reducing methanogenic archaea abundance in the studied flooded paddy soil. Methanogens and methanotrophs regulate CH_4_ emissions in paddy soils [196]. Feng et al. [197] and Qin et al. [198] found significant decreases in CH_4_ emissions by biochar addition and explained the result by increases in methanotrophic bacteria biodiversity and abundance. The addition of biochar to paddy soil reduces soil bulk density and enhances aeration, thereby suppressing methanogenic activity. This reduction in bulk density is attributed to biochar’s high porosity [199]. As a nutrient‐rich amendment [200], biochar stimulates rice root growth [201], leading to increased oxygen secretion [202, 203]. Enhanced oxygen availability further inhibits methanogens and their activity. Kim et al. [204] found that biochar application not only boosted rice yield but also reduced CH_4_ emissions by improving soil aeration and oxygen supply, thereby suppressing methanogenesis. (2) Electron Transfer and Methane Suppression: Biochar acts as an electron acceptor, facilitating alternative microbial pathways that compete with methanogenesis. Biochar contains quinone, phenolic and other redox‐active groups that can accept electrons from microbial metabolism, especially from anaerobic respiration [205]. These functional groups undergo reversible oxidation and reduction, influencing microbial electron transfer. Theoretically, carbonyl and quinone moieties in biochar can function as electron acceptors, facilitating CH_4_ consumption in paddy soils. Zhang et al. [206] reported that the quinone (C=O) structure in biochar contributes to anaerobic CH_4_ oxidation. This process can account for up to 50% of total CH_4_ consumption in wetlands [207]. Therefore, biochar may help mitigate CH_4_ emissions partly by enhancing anaerobic CH_4_ oxidation through its electron‐accepting capacity in paddy soils. (3) Nutrient and pH Modification: Biochar can increase soil pH, which inhibits methanogenic archaea that prefer acidic conditions. The alkaline nature of biochar is mainly due to the ash content [148]. Weber and Quicker [208] observed a large decrease in methanotrophic activity when soil pH decreases from 6.3 to 5.6. Generally, at higher pyrolysis temperatures, the biochar produced is higher alkaline, and hence, its application significantly promotes the activity of methanotrophic in acid paddy soil [209]. (4) Reduced Substrate Availability: Biochar adsorbs DOC, decreasing the availability of methanogenic precursors like acetate and hydrogen. Soil DOC serves as a key substrate for methanogens and plays a crucial role in CH_4_ production [196]. Yu et al. [184] found that applying hen biochar to paddy fields reduced soil DOC, likely due to adsorption within the biochar pores. Zheng et al. [210] reported that biochar amendment decreased DOC content by 52% and 71% at application rates of 20 and 40 t ha^−1^, respectively (Table 4).

**Table 4:** Effects of biochar on methane (CH_4_) sequestration.

Theme/mechanism	Main findings	Benefits/implications	Citations
CH_4_ reduction in forest and plantation soils	CH_4_ emissions reduced with 10% w/w chicken manure biochar; increased CH_4_ uptake in chestnut plantations regardless of rate	Demonstrates biochar effectiveness across forest and perennial systems	[185, 210]
pH enhancement promoting methanotrophs	Biochar increases soil pH, stimulating methanotrophic growth and CH_4_ oxidation	Improves microbial CH_4_ consumption and reduces emissions	[186, 187]
Soil structural improvement (bulk density↓, porosity↑)	Increased soil porosity and reduced bulk density enhance CH_4_ oxidation and diffusion	Supports microbial CH_4_ uptake under better‐aerated conditions	[188, 189]
Chemical inhibition of methanotrophs	Certain biochar‐derived compounds affect methanotroph communities	Identifies chemical pathways influencing CH_4_ dynamics	[190]
Paddy soil CH_4_ reduction	Bamboo and straw biochar (2.5%) reduced CH_4_ emissions by 51%–91% in 49 days	Strong mitigation potential in flooded rice systems	[191]
Global evidence from meta‐analysis	Biochar reduces CH_4_ emissions, improves SOC and increases crop yield, especially in paddy soils and acidic environments	Confirms global‐scale CH_4_ mitigation effectiveness	[187, 193]
Rice husk biochar (RHB) effects	RHB improved SOC and rice carbon accumulation; reduced CH_4_ emissions by 50%–60% compared to FYM	Enhances soil quality while lowering greenhouse gas emissions	[193]
Mangrove wood biochar in rice systems	Biochar reduced cumulative CH_4_ emissions by 21%–25% across two seasons; biochar + fertilizer > fertilizer alone	Effective for tropical lowland rice systems	[194]
Corn residue biochar under drip‐irrigated maize	At 15–30 t ha^−1^, biochar reduced CH_4_ and N_2_O emissions, increased SOC and lowered GWP	Valuable mitigation tool in semiarid maize production	[195]
Enhanced aeration suppressing methanogenesis	Biochar increases O_2_ diffusion, reducing methanogenic archaea and increasing methanotroph abundance	Limits CH_4_ production and enhances oxidation in flooded soils	[170, 196–200]
Electron‐accepting properties (quinone groups)	Biochar acts as an electron acceptor, promoting anaerobic CH_4_ oxidation; quinone groups reduce CH_4_ by facilitating microbial pathways	Provides alternative pathways for microbial metabolism competing with methanogenesis	[208]
pH modification and nutrient effects	Alkaline biochar (high‐temperature pyrolysis) boosts methanotrophic activity in acidic soils	Particularly effective in acidic paddy soils	[148, 184, 209]
Reduced substrate availability (DOC adsorption)	Biochar adsorbs dissolved organic carbon (DOC), reducing precursors for methanogenesis	Limits CH_4_ production at microbial substrate level	[210], 225]

## 2. Conclusion

This review revealed that GHG emissions, including CO_2_, CH_4_ and N_2_O, detrimentally affect crop productivity, posing a serious threat to global food security. These gases trap heat in the Earth’s atmosphere, leading to global warming and climate change, ultimately impacting agriculture and crop yields. Decreased crop productivity due to climate change can have far‐reaching economic and social impacts. Reduced yields may lead to food shortages, increased food prices and economic instability, affecting both farmers and consumers, particularly in vulnerable regions. Biochar aids in mitigating atmospheric CO_2_ by sequestration of C (for it is difficult for the sequestered carbon to be released as CO_2_, making this a good method for carbon sequestration). Biochar can positively influence soil physical properties, such as reducing bulk density and enhancing soil moisture, potentially leading to a decrease in soil N_2_O emissions. The decrease in soil N_2_O emissions was due to enhanced oxygen levels in the soil by biochar through improved aeration. Biochar has been utilized to mitigate methane (CH_4_) emissions. The reduction in CH_4_ due to biochar can be linked to the inhibitory effect of biochar chemicals on soil methanotrophs.

Biochar’s potential as an agent of GHG sequestration lies in its ability to effectively capture and store carbon while promoting soil health and reducing emissions of other potent GHGs. When integrated into sustainable land management practices, biochar can play a vital role in mitigating climate change and fostering a more sustainable and resilient agricultural system. However, further research and widespread adoption are essential to fully realize the potential of biochar on a global scale.

### 2.1. Potential Areas of Future Research

Future studies should explore the economic feasibility of integrating biochar into agricultural systems by assessing the cost‐effectiveness of its production and application relative to potential benefits, including increased crop yields, carbon sequestration and reduced emissions. In addition, research should examine the development and implementation of policies and regulatory frameworks that drive the adoption of biochar in agriculture, as well as assess the potential barriers and incentives at local, national and international levels for the integration of biochar into sustainable land management practices.

## Funding

This research received no external funding.

## Conflicts of Interest

The authors declare no conflicts of interest.

## Data Availability Statement

The authors have nothing to report.
